# Efficacy and Safety of Direct Oral Anticoagulants in Venous Thromboembolism Compared to Traditional Anticoagulants in Morbidly Obese Patients: A Systematic Review and Meta-Analysis

**DOI:** 10.7759/cureus.14572

**Published:** 2021-04-20

**Authors:** Anjan Katel, Madan Aryal, Arun Neupane, Rohit Gosain, Ranjan Pathak, Yashoda Bhandari, Peter Kouides

**Affiliations:** 1 Department of Internal Medicine, Kathmandu University School of Medical Sciences, Dhulikhel, NPL; 2 Department of Medicine, Enloe Medical Center, Enloe Regional Cancer Center, California, USA; 3 Department of Nursing and Critical Care, Alta Bates Summit Medical Center, California, USA; 4 Department of Medicine, Roswell Park Comprehensive Cancer Institute, New York, USA; 5 Department of Oncology, Nepal Medical College Pvt. Ltd., Kathmandu, NPL; 6 Department of Nursing, University at Buffalo, New York, USA; 7 Department of Hematology/Oncology, Rochester General Hospital, Rochester, USA

**Keywords:** morbid obesity, doacs, vte prophylaxis, vitamin k antagonists, non-vitamin k oral anticoagulant

## Abstract

Background

Randomized clinical trials comparing the efficacy and safety of direct oral anticoagulants (DOAC) with vitamin K antagonist (VKA) or low molecular weight heparin (LMWH) for the treatment of venous thromboembolism (VTE) generally exclude patients who are morbidly obese (body mass index ≥ 40 kg/m^2^ or weight ≥ 120 kg). Recently, smaller studies have compared DOACs with warfarin or low molecular weight heparin (LMWH) in morbidly obese patients with VTE. We aim to systematically review and do a meta-analysis of the studies that directly compared DOACs with VKAs or LMWH in morbidly obese patients.

Methods

Studies comparing DOAC with warfarin or LMWH in patients with acute VTE were identified through electronic literature searches of MEDLINE, EMBASE, Scopus, clinicaltrials.gov, and the Cochrane Library up to March 2020. The primary efficacy outcome was recurrent VTE and the primary safety outcome was major bleeding as defined by the International Society on Thrombosis and Haemostasis (ISTH) guidelines. Study-specific odds ratios (OR) were calculated and combined using a random-effects model meta-analysis.

Result

Five studies were identified. Recurrent VTE occurred in 95 of 3207 (2.96%) patients in the DOAC group and 81 of 3181 (2.54%) patients in the VKA and LMWH group (OR: 1.17; 95% CI 0.87 to 1.59, p=.30). Major bleeding occurred in 63 of 3316 (1.89%) patients in the DOAC group, and 83 of 3259(2.54%) patients in the VKA or LMWH group (OR: 0.74; 95% CI: 0.53 to 1.03, p=.08). Sensitivity analysis comparing factor Xa inhibitors apixaban and rivaroxaban to warfarin also yielded consistent findings.

Conclusion

DOACs showed similar efficacy and safety in the prevention of recurrent VTE risk and major bleeding events in morbidly obese patients when compared to warfarin/LMWH. Our study underscores the need for further modifications of therapy to reduce the high VTE recurrence rate irrespective of whether the patient is on a DOAC or VKA. This might be possible through a very large multi-institutional randomized clinical trial.

## Introduction

Direct oral anticoagulants (DOACs), such as apixaban and rivaroxaban, are becoming preferred agents in the treatment of acute venous thromboembolism (VTE) as compared with vitamin K antagonists (VKA) due to numerous advantages, including fewer monitoring requirements, fewer dietary restrictions, and fixed dosing [1-2]. Data from clinical trials in patients with acute VTE and non-valvular atrial fibrillation (NVAF) have shown that DOACs have similar efficacy in the treatment of VTE as compared with VKAs with a similar or lower bleeding risk [3].

Obesity is a pro-inflammatory and prothrombotic state [4] and an independent risk factor for VTE, increasing the risk of VTE by two to six-fold [5]. Due to alterations in pharmacokinetic parameters including volume of distribution, half-life, and clearance, anticoagulant medication doses often require adjustments in obese patients [6-7]. Although there are limited data evaluating the efficacy of DOACs in non-morbidly obese patients (body mass index (BMI) <40 kg/m2) in the treatment of VTE, less information exists to guide the treatment of morbidly obese patients (BMI ≥40 kg/m^2^ or weight ≥120 kg) [8] since they were underrepresented in clinical trials. Furthermore, the adequacy of fixed-dose anticoagulation in this population has been questioned due to concerns about efficacy [9]. The International Society on Thrombosis and Haemostasis (ISTH) recommends avoiding DOACs in morbidly obese patients and closely monitoring plasma drug levels if used [7]. Similarly, laboratory monitoring of anti-Xa while using LMWH in morbidly obese populations has been suggested [10]. However, plasma drug monitoring for DOACs may not be readily available, thus limiting the use of DOACs in these patients despite patient preferences.

We, therefore, sought to perform a systematic review and meta-analysis to examine the efficacy and safety of DOACs in morbidly obese patients in the treatment of VTE in the reported literature to date.

## Materials and methods

The Preferred Reporting Items for Systematic Reviews and Meta-Analyses (PRISMA) statement for reporting systematic reviews as recommended by the Cochrane Collaboration was followed in this systematic review [11].

Literature search

A systematic literature review using MEDLINE, EMBASE, Cochrane CENTRAL, Scopus, and clinicaltrials.gov as performed from each database inception to March 2020.

The search was restricted to English-language publications and was performed applying all potential synonyms of four broad themes: “direct oral anticoagulants,” “warfarin,” “factor Xa inhibitors,” and “low molecular weight heparins,” and combined using the Boolean operator “AND.” References from review articles, editorials, and conference publications were hand-searched and cross-referenced to ensure a comprehensive search.

Study selection

We included all studies that evaluated the treatment of acute VTE in adult (age ≥18) obese patients that compared rivaroxaban, apixaban, edoxaban, or dabigatran with VKA or LMWH and reported the incidence of VTE or acute bleeding in the first 12 months.

Studies of DOACs in NVAF were not included. In addition, studies in VTE that did not report VTE and major bleeding outcomes separately were also excluded [12]. The study by Coons et al. was not included due to a lack of separate data on morbidly obese patients [9]. Studies that evaluated both VTE and NVAF patients, but did not report data separately were also excluded [13-14]. Two studies were not included, as both intervention and control arms were DOACs [12,15]. Prior studies comparing DOACs in obesity have been listed in Table 1.

**Table 1:** Baseline characteristics of included studies A, apixaban; ARR, absolute risk reduction; CRNMB, clinically relevant non-major bleeding; NS, not specified; R, rivaroxaban; VA, Veterans Affairs; W, warfarin; PPPY, per patient per year, DOAC: directly acting anticoagulants (including apixaban, rivaroxaban, and dabigatran), TT: traditional therapy (including warfarin and low molecular weight heparin)

	Kushnir et al,2019	Perales et al,2019	Spyropoulos et al, 2019	Sa et al, 2019	Quan et al, 2020
Source type	Journal article	Journal article	Journal article	Journal article	Journal article
Study design	Single-center, retrospective analysis of chart data Montefiore Medical Center (Bronx, NY, USA)	Retrospective chart review at 2 academic medical centers	Retrospective 1:1, propensity score-matched cohort study using 2 US claim database	Retrospective, single-center cohort study in London (Canada)	Sub-study of retrospective chart review at Canadian institutions
Comparison made	A Vs R VS W	R Vs W	R vs W	DOAC vs W	DOAC vs TT
Inclusion criteria	BMI ≥40, ≥18 years old, Included AF and VTE (Only VTE data taken)	BMI>40, >120kg Included AF and VTE (only VTE data taken)	ICD 9/ICD 10 diagnosis code for morbid obesity. Included VTE patients	BMI ≥ 30 kg/m^2^ versus < 30 kg/m^2^ Weight ≥120 kg vs. <120 kg(Only data from weight ≥120 kg included)	Weight >120kg, VTE only
Mean age, years	53 A	56+/-14 R	53 R	NS	53 DOAC
	52 R	55+/- 15 W	53 W		52 TT
	58 W	NS	NS		
Female sex, %	74.4 A	48 R	60.5 R	NS	34 DOAC
	65.7 R	44.5 W	60.2 W		35.9 TT
	70.6 W				
Mean BMI (kg/m^2^)	43.3 A	45 R	NS	NS	NS
	43.7 R	44W			
	45.3 W				
Mean weight, kg	NS	133 R, 134 W	NS	NS	138 DOAC
					142 TT
Medications, %	NS		NS	NS	NS
Antiplatelet agents		ASA: 34/84 R, 35/92 W Clopidogrel: 4/84 R, 8/92 W NSAID: 9/84 R, 3/92 W			
Comorbidities	Charlson Comorbidity Index 0 A 1 R 2 W	3 R, 3 W	1.2 R, 1.2 W	NS	
Malignancy	NS	3/84 R, 7/92 W	211/2890, 199/2890		3/109 DOAC, 16/78 TT
Chronic kidney	NS	0/84 A, 10/92 W	182/2890 (6.3%),R, 203/2890 (7.0%) W		
Total duration of study, mo Follow-up	163 days (A), 217 days (R), 206 days (W)	12 months	10 months R, 10.5 months W	1 year	1 year
Study groups (total no. of patients in each group)	3 groups	2 groups	2 groups	2 groups	2 groups
	47 A	84 R	2890 R	71 DOAC	DOAC 109
	152 R	92 W	2890 W	62 W	TT 78
	167W				
Primary outcome	Recurrent VTE, Major bleeding	VTE recurrence Mortality	VTE recurrence Major bleeding	VTE recurrence	Overall rate of recurrent VTE
Secondary outcome	Composite bleeding	Length of stay and bleeding complications	Major Bleeding risk Healthcare resource utilization and costs	Major bleeding	Rate of recurrent VTE on and off anticoagulation therapy, dosing regimens used, duration of therapy, bleeding events, and proportions of patients prescribed a DOAC only vs TT only vs having therapy switches.
Recurrent VTE, %	2.1 A		Risk- R 16.8 % (485/2890)	2.81 DOAC	0.006 events per patient year
	2.0 R	4.2 R	Risk- W 15.9% (459/2890)	0 W	
	1.2 W	6.4 W	Actual- R-0.02 PPPY Actual- W-0.03 PPPY		
Major bleeding, %	2.1 A	6.3 R	1.8 R	1.4 DOAC	3.6 DOAC
	1.3 R				
	2.4 W	3.2 W	2.5 W	1.6 W	3.8 TT
Nonmajor/minor bleeding, %	NS (composite bleeding) 10.7 A 9.8 R 16.4 W	NS	NS	NS	NS
Study outcomes reported at	Within 6 months	Within 12 months	Time to first major bleeding event- R 69 days (mean 83), W-77 days (mean 79)	Within 1 year	Within 12 months

In case of multiple publications resulting from the same study, we included data from the most recent publication. Two investigators (R.G. and A.K.) screened and retrieved relevant articles and excluded irrelevant studies. Relevant data were extracted by two investigators (R.G. and A.K) and checked by two other authors (A.N. and Y.B.). Two additional investigators (M.R.A. and R.P.) participated in the review process when uncertainty about eligibility criteria arose.

Outcomes and data extraction

The primary efficacy outcome of interest was the rate of recurrent VTE (composite of any recurrent deep vein thrombosis or pulmonary embolism) in the first 12 months; the primary safety outcome was major bleeding.

Recurrent VTE was defined as positive imaging findings on ultrasound Doppler or computed tomography. Major bleeding was defined as bleeding events requiring intervention or transfusion, cardiac tamponade, or pericardial effusion requiring drainage, retroperitoneal bleeding, intracranial bleed, massive hemoptysis, hemithorax, bleeding requiring an extra hospital stay, and patients requiring hospitalization as per the ISTH guidelines [16]. Rates of minor bleeding were not included due to significant missing data.

The following details from each study were extracted and tabulated: study design, modes of comparison, mean age, percentage of female population, mean BMI, mean weight, medications, comorbidities number of patients with acute VTE, estimated recurrent VTE risk, estimated major bleeding risk, duration of anticoagulation, and follow-up period.

Risk of bias

We assessed the risk-of-bias using a modified Newcastle-Ottawa Quality Assessment Scale for retrospective studies [17].

Statistical analysis

Outcomes from the individual studies were aggregated with RevMan (version 5.3, Cochrane Collaboration, Oxford, United Kingdom) applying the Mantel-Haenszel test. Odds ratios (ORs) and 95% confidence intervals (CIs) were estimated using a random-effects method to account for the presence of variability among the studies. The I^2^ statistic was used to assess heterogeneity. Two-tailed p-values <.05 were considered to indicate statistical significance.

## Results

Study selection

**Figure 1:**
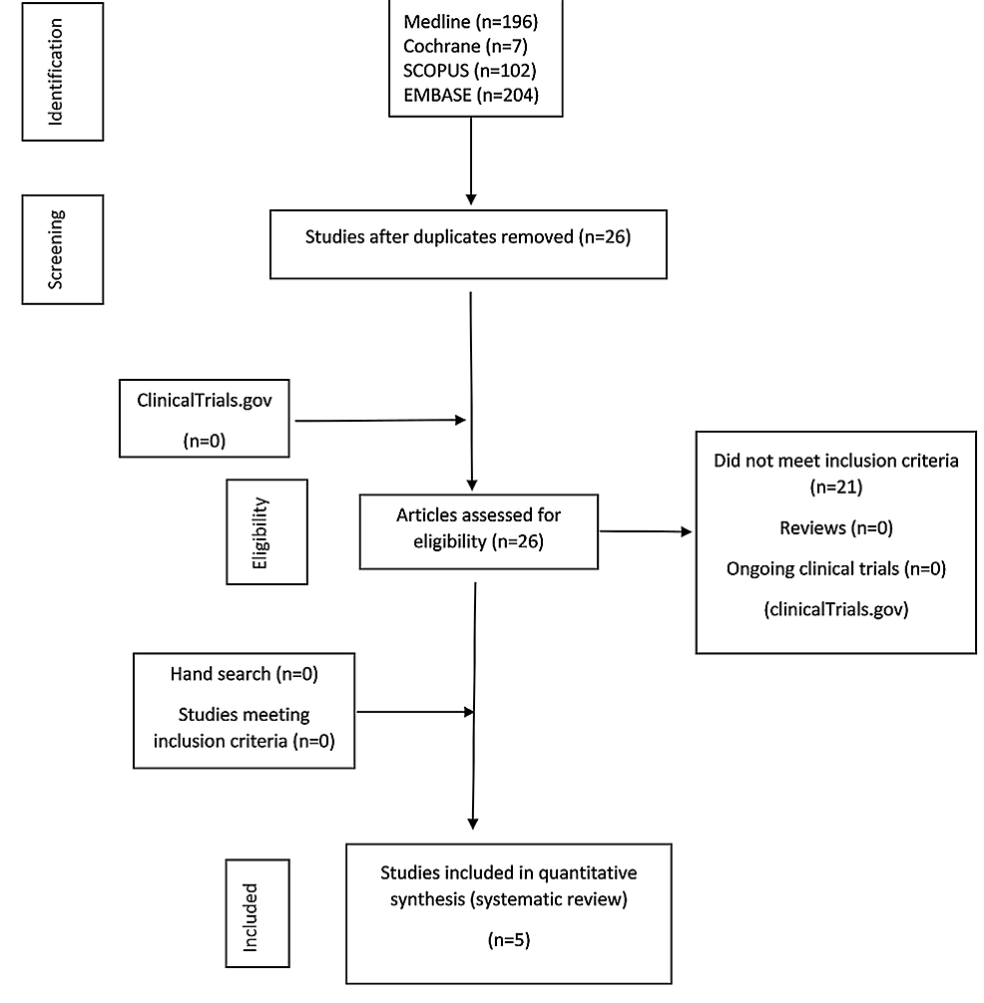
Flowchart describing the systematic search and study selection process

The literature search identified 509 unique references. After a full-text review of 26 articles, we identified five observational studies for qualitative and quantitative analysis (Figure 1).

Study characteristics

Five relevant studies with 6575 patients were included. In studies that included both NVAF and VTE patients, data were extracted for VTE patients only [18-19]. In the study by Quan et al., the analysis was limited to major bleeding rates, as data on VTE recurrence were not reported [20]. For the number of VTE events in the study by Spyropoulos et al., we considered VTE events as diagnosed from imaging studies or VTE as primary inpatient diagnosis (Table 2) [21]. In the study by Sa et al., only patients with weight ≥20 kg were included [22]. Analysis of quality found scores of 7 of 9 for all reviewed studies, with each losing points for reporting of the adequacy of follow-up (Table 3).

**Table 2:** Prior studies comparing DOACs in obesity VTE, venous thromboembolism, AF, atrial fibrillation, BMI, body mass index; PE, pulmonary embolism; A, apixaban; NS, not specified; R, rivaroxaban; W, warfarin; TIA: transient ischemic attack; DOAC: directly acting anticoagulants (including apixaban, rivaroxaban, and dabigatran), TT: traditional therapy (including warfarin and low molecular weight heparin (LMWH))

Study and study type	Drugs compared	Total patients	Weight	Recurrent VTE events	Major bleeding events	Included for the study? If no, why?
RECOVER 1 and 2 Randomized double-blind trial with subgroup analysis based on weight	a. Dabigatran and b. Warfarin for VTE	N=5107	Subgroup weight > 100 kg (N=832)	No interaction between body weight and primary endpoints (recurrent VTE and deaths)	NS	No. Because there is no separate data for morbidly obese patients
AMPLIFY randomized double-blind trial with subgroup analysis based on weight	a. Apixaban and b. Warfarin for VTE	N= 5400	Subgroup weight > 100 kg (N=1017)	No interaction between body weight and primary endpoints (recurrent VTE and deaths)	No interaction between body weight and major bleeding	No. Because there is no separate data for morbidly obese patients
EINSTEIN-PE Randomized, open-label, with subgroup analysis based on weight	a. Rivaroxaban and b. Warfarin for PE	N=4833	Subgroup weight>90 kg (N=1355)	No interaction between body weight and primary endpoint (recurrent VTE)	No interaction between body weight and major bleeding	No. Because there is no separate data for morbidly obese patients
EINSTEIN-DVT Randomized, open-label, with subgroup analysis based on weight	a. Rivaroxaban and b. Warfarin for VTE	N=3499	Subgroup weight > 90 kg (N=969)	No interaction between body weight and primary endpoint (recurrent VTE)	No interaction between body weight and major bleeding	No. Because there is no separate data for morbidly obese patients
HOKUSAI VTE Randomized, double-blind with subgroup analysis based on weight	a. Edoxaban and b. Warfarin for VTE	N=4921	Subgroup weight > 100 kg (N=1265)	No interaction between body weight and primary endpoint (recurrent VTE)	No interaction between body weight and major bleeding	No. Because there is no separate data for morbidly obese patients
Kushnir et al. Single-center, retrospective analysis	a. DOAC: Rivaroxaban or Apixaban and b. Warfarin for either VTE or AF	N=795 For VTE: R:152 A:47 W:167	BMI>40 kg/m^2^	For VTE: Similar incidence of recurrent VTE between Apixaban (2.1%), rivaroxaban (2%), and warfarin (1.2%) cohort	For VTE: Similar incidence of major bleeding events in 2 cohorts	Yes
Syropoulos et al. Retrospective study of 2 databases	a. Rivaroxaban and b. Warfarin for VTE	N=5780 R: 2890 W: 2890	BMI>40 kg/m^2^	Similar incidence of recurrent VTE between Rivaroxaban (16.8%) and warfarin (15.9%)	Similar incidence of major bleeding	Yes. But only patients that fulfilled the definition of recurrent VTE as VTE events as diagnosed from imaging studies or VTE as primary inpatient diagnosis were taken.
Coons et al. Retrospective study	a. DOAC: Apixaban or Rivaroxaban or Dabigatran and b. Warfarin for VTE	N=1840 DOAC: 632 W:1208	300kg>Weight >100kg	No significant difference between DOAC (6.5%) and warfarin (6.4%) in the prevention of recurrence of VTE	No significant difference in major bleeding events	No. Because there is no separate data for morbidly obese patients
Perales et al. Retrospective review at 2 academic medical centers	a. Rivaroxaban and b. Warfarin for VTE and AF	N=176 In VTE patients: R: 47 W: 62	BMI>40 kg/m^2^ Weight> 120 kg	In VTE patients: No significant difference in the recurrence of VTE in rivaroxaban (4%) and warfarin (6%)	In VTE patients No significant difference in bleeding events in rivaroxaban (6%) and warfarin (3%)	Yes
Kalani et al. Retrospective study	a. DOACs: Apixaban or Rivaroxaban or Dabigatran b. Warfarin for VTE and AF	N=180 No separate data for VTE and AF patients DOAC:90 W:90	BMI>40 kg/m^2^ Weight> 120 kg	For combined VTE and AF: No significant difference in the primary outcome (composite of stroke, TIA, VTE, PE) in both arms.	Similar bleeding events in DOAC and warfarin group	No. Because there is no separate data for VTE and AF patients
Quann et al. Retrospective sub-study	a. DOAC: Apixaban or Rivaroxaban or Dabigatran and b. TT: LMWH or warfarin	N=187 DOAC: 109 TT: 78	Weight> 120 kg	Overall rate of recurrent VTE out to 1 year was 0.006 events/ patient-year	Similar bleeding events in DOAC (8.3%) and TT (11.5%)	Yes. But only bleeding event taken because the primary outcome was the overall rate of recurrent VTE.
Tittl et al. Prospective Dresden registry study	DOAC in different BMI groups for VTE and AF	N=3432	6 groups stratified according to BMI (in kg/m^2^) a. <18.5 b. 18.5-24.9 c. 25-29.9 d. 30-34.9 e. 35-39.9 f. >40	Thromboembolic rate: In BMI> 40: 0.49 events/100 patient-years	Similar major bleeding rates in between those with BMI>30 and <30 kg/ m^2^	No. Because it compared the clinical outcome of DOAC in different BMI groups
Aloi et al. Retrospective analysis	DOAC for VTE in patients with weight ≥ 120kg compared to that with <120 kg	N=1196	Two groups according to weight a. ≥ 120kg b. <120 kg	No significant difference in the recurrence of VTE in both groups	NS	No. Because it compared clinical outcome of only DOAC in 2 different weight group
Netly et al. Retrospective study	DOAC in different BMI groups for AF, VTE, and VTE chronic prophylaxis	N=113	3 groups stratified according to BMI (in kg/m^2^): a. <30 b. 30-40 > 40	No statistically significant differences in 3 groups. BMI> 40 kg /m^2^ had highest thrombotic events.	No statistically significant differences in 3 groups. BMI> 40 kg /m^2^ had lowest bleeding events.	No. Because it compared the clinical outcome of only DOAC in different BMI groups
Sa et al. Retrospective study	a. DOAC: Rivaroxaban, Apixaban and edoxaban b. Warfarin	N=133 DOAC: 71 W: 62	a.120>Weight≥ 120kg b. 30 kg/ m^2 ^>BMI ≥30 kg/m^2 ^only weight ≥120kg selected	No significant difference in the recurrence of VTE in both groups	Similar major bleeding rates between two groups	Yes. But only data from the weight group ≥120 kg.

**Table 3:** Modified Newcastle-Ottawa Quality Assessment Scale for retrospective studies mos: Months

Included studies	Selection				Comparability	Outcome			Classification
Study (Year)	Representativeness of exposure group	Representativeness of non-exposed groups	Ascertainment of exposure	Determination that outcome not present initially	Comparison of cohorts	Assessment of outcome	Long enough follow-up?	Adequacy of follow-up?	
Kushnir (2019)	Yes	Yes	Secure record	Yes	Yes	Reported	Yes (3 mos and 15 mos)	No (Lost to follow-up not reported)	7
Parales (2019)	Yes	Yes	Secure record	Yes	Yes	Reported	Yes (12 mos )	No (Lost to follow-up not reported)	7
Spyropoulos (2019)	Yes	Yes	Secure record	Yes	Yes	Reported	Yes (10 mos)	No (Lost to follow-up not reported)	7
Quan (2019)	Yes	Yes	Secure record	Yes	Yes	Reported	Yes (12 mos)	No (Lost to follow-up not reported)	7
Sa (2020)	Yes	Yes	Secure record	Yes	Yes	Reported	Yes (12 mos)	No (Lost to follow-up not reported)	7

Recurrent VTE

**Figure 2:**
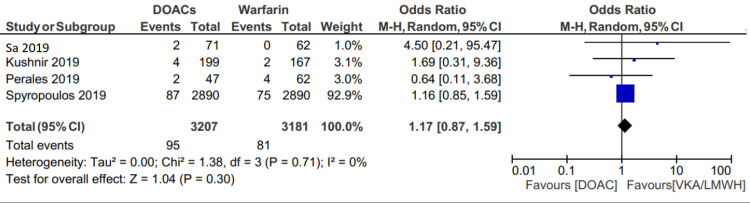
Forest plot showing the comparison of recurrent VTE between two groups VTE, venous thromboembolism

**Figure 3:**
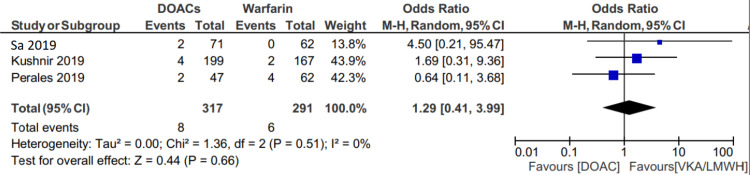
Forest plot showing a comparison of recurrent VTE between two groups excluding the study by Spyropoulos et al. VTE, venous thromboembolism

Recurrent VTE occurred in 95 of 3207 (2.96%) patients in the DOAC group and 81 of 3181 (2.54%) patients in the VKA and LMWH group (OR: 1.17; 95% CI 0.87 to 1.59, p=.30) (Figure 2). Slightly higher rates of VTE were mainly driven by the Spyropoulos et al. study [21], possibly driven by the fact that 7% of their population had malignancy and another 7% had chronic kidney disease. Sensitivity analysis performed excluding this study found VTE recurrence in DOACs of 2.52% and VKA/LMWH of 2.06% (OR: 1.29; 95% CI: 0.41 to 3.99, p=.66) (Figure 3).

Major bleeding

**Figure 4:**
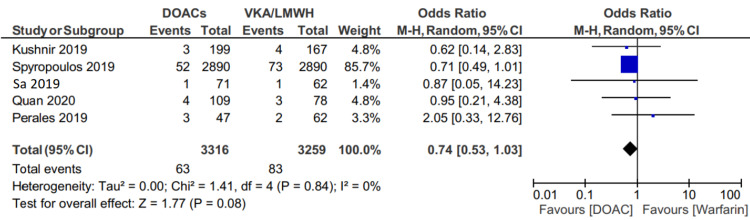
Forest plot showing a comparison of major bleeding between two groups

Major bleeding occurred in 63 of 3316 (1.89%) patients in the DOAC group, and 83 of 3259(2.54%) patients in the VKA or LMWH group (OR: 0.74; 95% CI: 0.53 to 1.03, p=.08) (Figure 4).

Although the majority of the included studies compared factor Xa inhibitor (apixaban and rivaroxaban) with warfarin, the study by Quan et al. [20] also included dabigatran with other DOACs. Given that the Quan et al. study also included dabigatran, we performed a sensitivity analysis excluding this study to isolate the Xa inhibitors and found similar results.

**Figure 5:**
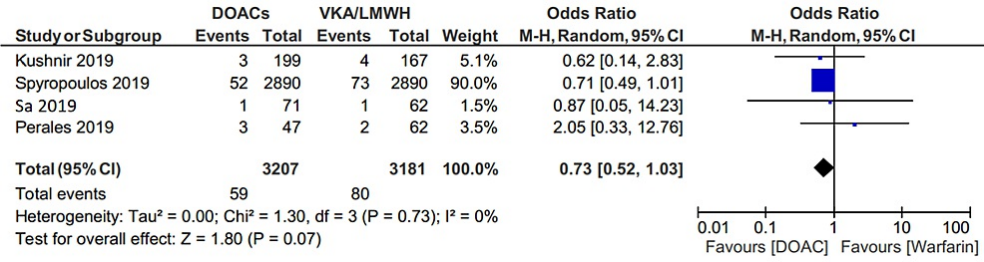
Forest plot showing a comparison of major bleeding between two groups excluding the study by Quan et al.

Additional sensitivity analysis comparing the factor Xa inhibitors apixaban, rivaroxaban, and edoxaban to warfarin showed similar rates of major bleeding (59/3207 (1.83%) patients in the factor Xa inhibitor group versus 80/3181 (2.51%) patients in the VKA group (OR 0.73; 95% CI 0.52 to 1.03, p=.07) (Figure 5).

## Discussion

In this study, we found a similar incidence of recurrent VTE and major bleeding with both DOACs and VKA/LMWH in morbidly obese patients. Our study is one of the few studies to date examining the safety and efficacy profiles of DOAC in these patients.

Our results are consistent with a prior meta-analysis looking at VTE prophylaxis in obese patients in a post-arthroplasty setting that compared DOACs to LMWH. Our data are also consistent with a prior meta-analysis of DOACs for the treatment of VTE in non-morbidly obese patients [23]. Although our findings are similar to another meta-analysis by Elshafei et al., our study has clear data presentation and summarization of the study and sensitivity analysis [24]. Our results also align with data from a study comparing DOACs with VKA in morbidly obese patients (BMI>40 kg/m^2 ^and weight>120 kg) with NVAF that showed similar efficacy in the prevention of VTE recurrence, systemic embolism, and a similar bleeding risk [14,18].

We notice that the rate of recurrent VTE in our study was higher in both arms (2.9%, for DOACs, 2.5% in VKAs) than reported in the prior DOACs study in non-obese patients, which presumably were randomized trials. We hypothesize that this may be related to an increased risk of recurrent VTE in obese individuals [25]. There could be a higher incidence in the studies included here because the inclusion criteria were less stringent than in randomized trials and thus older and/or sicker patients were in the cohort studies. This study underscores the need for further modifications of therapy to reduce the high VTE recurrence rate irrespective of whether the patient is on a DOAC or VKA. In the case of DOACs, studying dosing continuously at 15 mg bid of rivaroxaban or 10 mg bid of apixaban beyond the respective “lead-in” periods in morbidly obese patients would seem worthy of a clinical trial but given the relatively low recurrence rate, this would have to be a very large multi-institutional randomized clinical trial. Given that our study includes only patients with BMI ≥40 kg/m^2^, our data helps understand the effect of extremes of body weight on the efficacy of DOACs. Further studies are needed to determine if there is a “ceiling effect” precluding efficacy, possibly at a BMI of >60 kg/m^2^, as these patients were under-represented in our study.

In terms of safety data, we observed a similar lower rate of major bleeding with DOACs as compared with VKA or LMWH in morbidly obese patients. Previous studies examining the pharmacokinetic attributes of rivaroxaban have found a similar peak concentration, volume of distribution, and half-life between patients who weighed more than 120 kg and those who weighed between 70 and 80 kg [26]. However, in patients taking apixaban, higher body weight (>120 kg and BMI ≥30 kg/m²) was associated with a lower mean peak concentration, higher volume of distribution, lower drug exposure, and shorter mean half-life compared with normal weight (65-85 kg and BMI ≤30 kg/m²) [27]. These differences in pharmacokinetic properties by body weights do not seem to be consistent across various DOACs and, therefore, the clinical implications also remain unclear. Finally, although lower bleeding rates have been reported in prior studies with DOACs in major studies specifically with apixaban [23], we were unable to examine bleeding rates separately for each DOACs.

Given that some clinicians are already using DOACs, even in obese and morbidly obese patients, after a careful discussion of risk-benefit profiles with patients [4], our data give additional credence to this practice. Although only randomized controlled trials can make a definitive conclusion on the safety and efficacy of DOACs versus VKA/LMWH, these trials are unlikely to be conducted. Therefore, our data represent the best available evidence to guide clinical practice at this point.

Other strengths of our study include a large sample size with low heterogeneity (I^2^=0%) and a relatively uniform reporting of outcomes.

Limitations

The limitations of our study include the inclusion of non-randomized observationals that often have much large effect sizes than randomized trials, likely because of selection biases. In the warfarin arm, the time to therapeutic range was unknown due to missing long-term INR data. Similarly, included studies did not report measurement of DOAC levels to ensure therapeutic plasma drug levels. This could have potentially led to the under-dosing of DOACs. For example, a prior study found the prevalence of under-dosing with apixaban at 24.5% compared with 12.8% with rivaroxaban and 14% with dabigatran. Five percent of patients that were under-dosed with apixaban was related to patient body weight, unlike rivaroxaban and dabigatran where weight had no effect on under-dosing [28]. As we have noted, obesity may have a different impact on the pharmacokinetics of various DOACs. The approach of lumping patients treated with different DOACs as one group is unjustified, as it might obscure potential differences among the DOACs. Each DOAC should be treated as a separate group. Also, it is unclear what percentage of patients had adherence to the treatment in both arms. It is also unclear as to what percentage of included patients underwent bariatric surgery given data suggesting variable plasma drug levels of DOACs in bariatric surgery patients [29]. Finally, we were unable to ascertain the impact of comorbidities on both safety and efficacy outcomes. We were not able to determine what percentage of patients may have been on OCPs, had prior VTE events, or had cancer that was not captured by retrospective review.

## Conclusions

In this systematic review and meta-analysis of retrospective studies evaluating DOACs versus VKA/LMWH in morbidly obese patients, we demonstrate that both the safety and efficacy of DOACs might be similar to VKA/LMWH.
